# Teaching and learning lifestyle medicine during COVID-19: how has living during a pandemic influenced students’ understanding and attitudes to self-care and population health? A qualitative analysis

**DOI:** 10.1186/s12909-022-03590-6

**Published:** 2022-07-08

**Authors:** Christopher-James Harvey, Edward J. Maile, Ana Baptista, Richard J. Pinder

**Affiliations:** 1 Imperial College School of Medicine, London, UK; 2 School of Public Health, Imperial College London, London, UK; 3 Medical Education Research Unit, Imperial College London, London, UK

**Keywords:** Lifestyle Medicine, Public Health, Attitudes, Self-care, Constructivism, Health Behaviours, Pandemic

## Abstract

**Background:**

In 2019 a new Lifestyle Medicine (LM) module was introduced to the undergraduate medical curriculum at Imperial College London. Lifestyle Medicine is an emergent discipline which aims to tackle the increasing burden of non-communicable disease. Previous work has suggested that students value clinical teaching over traditional Public Health topics. Taking a constructivist view of learning, this paper assesses changes in medical students’ attitudes towards Public Health and LM in response to living through a pandemic. We then make suggestions as to how this lived experience might be useful in teaching LM, and discuss the interaction between teaching, behaviour, and experience with consideration of self-determination theories in learning.

**Methods:**

First-year medical students were surveyed at the end of their first year of teaching and asked if living during the COVID-19 pandemic had changed the value they place on LM and if so, how. Thematic analysis was conducted on responses representing 71% (*n* = 216) of the year group.

**Results:**

Four themes were defined in the data: acknowledging importance; impact on behaviour; health inequalities and the wider determinants; and promoting Public Health and prevention. These themes highlight the distinct levels through which the pandemic has had an impact: from personal behaviour to population health.

**Conclusions:**

This is the first study to look at the impact of living through a pandemic on attitudes to LM. Our results suggest that the pandemic has led to increased reflection on health behaviours. The lived-experience of COVID-19 may facilitate a better understanding of health inequalities and their impact, alongside the opportunities presented by effective LM interventions.

## Background

Lifestyle Medicine (LM) is an emerging integrative clinical discipline that is defined by the *American College of LM* as “the use of evidence-based lifestyle therapeutic intervention… to prevent, treat, and often reverse chronic disease”. Reflecting the global burden of non-communicable disease, LM education for medical students and doctors has been gaining traction over recent years [1] with increasing acceptance in medical practice [2] although it has not been without controversy [3].

In the past, the medical education evidence base has highlighted the phenomenon of medical students favouring more explicitly-relevant clinical skills compared to epidemiology or other Public Health learning [4, 5]. Prior to any clinical experience then, teaching LM may present a challenge: as the latter tends to focus on concepts rather than traditional biomedical knowledge, or clinical experience. Positively, more recent evidence has suggested that medical students find LM content interesting and ‘acceptable’ [6]. Yet the importance they place on it is unknown [7].

Lifestyle Medicine also shifts the paradigm of medicine from a traditional ‘doctor as expert’ model to a shared decision-making approach, where the patient is an active participant in their health and health outcomes. There are three key areas within our LM teaching:

1. Understanding the importance of sleep, nutrition, physical activity, and mental health protection on lifelong health outcomes.
2. Understanding the wider context in which a patient sits, and how this might affect their behaviours and barriers to changing behaviour
3. Using effective communication techniques to facilitate contextual understanding and promote behaviour change

To young people with no clinical experience these concepts may seem common sense, or woolly. This paper then sets out to assess the extent to which living during a pandemic provides a different context for this learning that changes attitudes to LM.

The rationale for this is situated within a constructivist view of learning [8]. Learners do not exist in an educational vacuum. They come to learning with prior knowledge, experience and socio-cultural perspectives and modelling. Constructivist approaches to education provide a lens through which to consider how all this informs learning, and the ways in which a learner extracts, or imports, meaning to/ from their learning. Indeed, it has been argued that the constructivist approach in medical education ‘makes more connections between different epistemological and pedagogical theories than any other (p. 1)’ [9]. Part of the learning process then is experiential learning or what Kolb calls ‘the transformation of experience (p.38)’ [10] The COVID-19 pandemic and the associated national lockdowns provides a natural, albeit awful, experiment that potentially brings to the fore the relevance of Public Health knowledge and skills in health protection and prevention and may enable students to contextualise the importance of LM and its allied disciplines.

## Methodology

Since 2019 all medical students at Imperial College School of Medicine (ICSM) in London have received core learning in their first two years of training through two innovative “Lifestyle Medicine and Prevention” (LMAP) modules that form 15% of all their learning and assessment in years 1 and 2 of the six-year undergraduate medical degree. Learning is designed through a model of flipped classroom [11] that leverages small group, clinician-led tutorials that seek to explore the interaction of wider health determinants on health behaviour and the inequalities that arise both in terms of opportunity and outcome. The module is built on firm, evidence-based principles of Public Health ethics and practice. It frequently draws on reflection [12] and causes students to examine both their own health behaviours, and those of their patients– whether positive or negative.

A qualitative study was designed to understand students’ perceptions of the impact of the pandemic on their views of LM.

### Context

This study was conducted three months into the first nationwide lockdown in England. It is important to understand what that meant for participants. At the time of the data being gathered students were not allowed to leave their homes except for 30 min daily exercise or other essential activities (i.e., food shopping). For many of our students this meant living in small, confined student halls. This was the last term of their first year at medical school. Daily deaths attributed to COVID-19 were approaching a nadir of around 130, after a peak in April of around 1,400 (data taken from the UK government website). This was a time of great uncertainty which saw the most vulnerable in our society disproportionately affected [13].

### Participants

First year undergraduate medical students at Imperial College School of Medicine (London, UK), were invited to participate at the end of their first year of medical school. Demographics of the sample can be seen in Table 1.

**Table 1:** Demographics of Participants

Age in years	Mean (Standard Deviation)	19.05 (0.78)
Gender (%)	Female	52.4
	Male	35.2
	Other	0.4
	Prefer not to say	0.7
	No Response	11.4
Ethnicity (%)	Asian	30.8
	White	21.2
	Mixed	18.7
	Other	11
	Black	2.2
	Prefer not to say	1.8
	No Response	14.3
Identifies as coming from a background where University isn’t the norm (%)	Yes	17.6
	No	66.3
	Prefer not to say	12.1
Holds a Previous Degree (%)	Yes	2.6
	No	86.1
	No Response	11.4

### Data collection

A questionnaire was purposefully designed to address the objectives mentioned above. It comprised mostly open-ended questions to allow students to reflect on their educational experience within the module, alongside Likert-style rating questions and a set of demographic characteristics.

### Procedure

At the end of their first academic year in June 2020, students were asked to complete a short questionnaire to facilitate reflection on their attitudes about and experiences of Lifestyle Medicine and Prevention learning. Students could opt in to have their responses to these open-ended questions saved for use in research. Their answers remained anonymous. Among other questions, students were asked how the pandemic was changing their attitude towards LM*.* Analysis of their responses to this question only is the focus of this paper.

### Data analysis

A thematic analysis to open text responses to the question ‘‘*Has the experience of COVID-19 changed the importance you place on Lifestyle Medicine? If so, how?’* was carried out in accordance with Braun and Clarke’s approach [14]. The thematic analysis was undertaken inductively and involved three coders. Coder one (C-JH) familiarised themselves with the data to code meaningful elements. Overarching themes were identified at the semantic level. More nuanced, granular themes were then reviewed and defined by coder two (AB). Finally, a validity check was conducted with a third coder (EJM), who independently coded a sample of responses across the themes and acted as an auditor. The analysis was carried out using NVivo software (QSR International Pty Ltd. (2020) NVivo (released in March 2020), https://www.qsrinternational.com/nvivo-qualitative-data-analysis-software/home.)

Of a potential 364 students, 295 consented to sharing their data and 260 provided a response to the question relating to their experience of the pandemic and perceptions of importance of LM. Forty-four students (16.9%) responded that the pandemic had not changed their views of LM. Three of these 44 explained that they attached significant importance to LM before the pandemic. No more can be extracted from these responses as the remaining participants did not provide free text responses. Therefore, thematic analysis was conducted on 216 free text responses.

### Ethics

The study was granted ethical approval from Imperial College London’s Faculty of Medicine Medical Education Ethics Committee (MEEC1920-181).

## Results

Four over-arching themes were identified. They can be seen in Table 2, along with definitions and sub-themes. They are expanded upon below, with selected quotes highlighting the content of each theme.

**Table 2:** Themes and sub-themes identified during thematic analysis and broad definitions

*Theme*	*Sub-Themes*	*Definition*
Acknowledging importance (Theme 1)	•Acknowledges importance for everyone •Acknowledges importance for patients •Acknowledges importance for self •Acknowledges importance for a specific aspect of wellbeing	This theme highlights students' increased awareness of the importance of health behaviours for their own and others’ health
Impact on behaviours (Theme 2)	•Intends to change behaviours •Has changed behaviour •Acknowledges difficulty in changing behaviour	This theme highlights the extent to which students have begun to change their health behaviours, or have a concrete plan to do so, due to the pandemic
Health inequalities and the wider determinants (Theme 3)	•Impact on specific social groups	This theme centres around the idea that COVID-19 has highlighted inequalities in health and health care and outcomes, and the role that LM plays in addressing these inequalities
Promoting Public Health and prevention (Theme 4)	•Prevention •Public Health risk	This theme reflects students’ increased awareness of the importance of Health interventions and health protection. This theme relates to population-health oriented interventions and policies

### Acknowledging importance (Theme 1)

This theme captures the sense that students described becoming more aware of the general importance of lifestyle for health and wellbeing conceptually but may not have made specific changes or plans to change their lifestyle.

*“Yes. Every aspect is addressed, staying indoors all day leads to poorer mental health, sick family members has led to financial strain, binge eating to cope with stress of exams and family health led to weight gain. Lifestyle medicine is more important now than ever"*

*“I think the pandemic gave me time and space to think about my lifestyle and how it impacts my health”*

*“Yes, in fact it was very interesting to study and learn about behaviour change during the pandemic because i could apply it to the real-life examples in a time where behaviour change is so critical amongst the general population”*

This theme breaks down into three sub themes: important for me; important for everyone and specific aspects of LM are important. The latter contained notable reference to how different health behaviours relate to mental health and wellbeing:

“*With the nation under lockdown it has made me value mental health greater and the importance of staying in good shape mentally and physically”*

*“Quarantine has been very challenging and made it easy to fall into bad eating and sleeping habits which then I started noticing were having an impact on my mood.”*

*“COVID-19 has made me realise how important exercise is not only to my physical but also mental wellbeing”*

*“When in lockdown…we need to focus more on sleep and mental health”*

*“I have been much more aware of how…things like exercise, sleep, nutrition, mental health have to do with my happiness…”*

Such statements suggest students can both recognise the contextual value of LM and relate such understanding back to their own experience as well and those of others in their wider communities.

### Impact on behaviours (Theme 2)

This theme highlights the extent to which students have begun to adapt their health behaviours, or have a concrete plan to do so, because of the pandemic. It also reflects changes in behaviour that align with the LM teaching they had received.

*“Definitely- I have been reconsidering my lifestyle a lot and want to keep healthy now more than ever”*

*“Healthy eating has always been important to me, but I appreciate taking care of my mental health more e.g. stepping away from social media, going for walks, sleeping earlier”*

*“The general concept of lifestyle medicine is integrating it into your day to day life. Certainly throughout the COVID-19 lockdown, I have attempted to stay fit and try to maintain an overall healthy lifestyle, which is due in part to the teaching that I had received throughout the LMAP module in my first year!”*

Students most reported physical activity as the tenet of lifestyle that they were particularly focussed on, and mental health and wellbeing as the health outcome they were most keen to maintain or improve.

### Health inequalities and the wider determinants (Theme 3)

This is a broader theme and touches on politics and justice in health. It relates to students learning the concepts of equality and equity in health. Underpinning this is the relationship between health, socioeconomic disadvantage, and deprivation. Some quotes suggest an increase in empathy towards those, who, outside of a pandemic, find themselves disadvantaged and unable to access health care.

*“It’s made me aware of the difficulty faced by people that don’t have the time, money and space to exercise and how much it can impact their health”*

There is also an expression of increased understanding of the role demographics play in health:

*“It shows me how different groups of people can be affected by COVID-19 e.g. BAME and how these are also be [sic] reflected in our LMAP teachings. Individuals with different levels of health, different backgrounds or ethnicities and [sic] more affected the likelihood of individuals contracting COVID as well as how it impacted them. So, I would say that I realised how LMAP is important and applicable”*

*“Yes, seeing how covid-19 has disproportionately affected people with different socio-economic backgrounds has shown me just how important lifestyle medicine is in preventing and treating disease.”*

### Promoting Public Health and prevention (Theme 4)

This theme highlights that living through the pandemic has made some students re-think the centrality of Public Health:

*“…public health has definitely been more at the forefront of the mind than ever before. The challenges and complexities of public health have become more apparent to us all”*

*“During covid-19 long term health problems such as diabetes and hypertension are much more dangerous and people with it must be protected much more”*

*“COVID-19 has made everyone make essential changes to their lifestyle, eg. increased efforts in personal hygiene and sanitation to decrease transmission and contraction of COVID, and these simple lifestyle changes have made me realise that it is quite important to practise these good habits even when there is no pandemic, as it is still protective against other illnesses and health problems”*

*“It definitely has, as one now notes the importance of good health and although COVID-19 is a transmissible disease and lifestyle medicine often concerns non-transmissible diseases, one does note that symptoms and prognosis might be altered if the individual was in better shape/ has better overall health (which is often heavily influenced by lifestyle medicine factors)”*

Within this theme responses highlight the recognition that certain risk factors for COVID-19 hospitalisation and mortality are modifiable: bringing forward the dividend of prevention. Collectively these responses suggest a shift in thinking, whereby the importance of lifestyle interventions not only benefit the individual in their day-to-day lives but offer long-term benefits for society. This shift from patients to communities is central to the delivery of population health thinking.

## Discussion

The findings of this paper provide an understanding of how the collision of emergent LM, wider Public Health teaching and a once-in-a-generation pandemic impact students attitudes to learning these topics. Students have described how their nascent understanding of health inequalities, preventive medicine and self-care have supported their behavioural response to the COVID-19 pandemic, and how this has impacted their attitudes to and understanding of LM. Taken together these findings suggest that Imperial’s new Lifestyle Medicine and Prevention learning has been advantageous for students and a potentially useful vehicle around which student health, LM skills, and population health teaching can be embedded.

While it is not possible to characterise a counterfactual scenario where either the LM teaching or the COVID-19 pandemic did not take place, these participant responses suggest the LM learning has provided knowledge and skills from which students can more positively engage with, and mitigate, the negative impacts from the pandemic.

While a minority of students stated that their experience of the pandemic had not affected their perspective on LM, the majority did. The government response to the COVID-19 pandemic in England included a series of lockdowns that affected every part of normal student life. At the same time, these lockdowns and the associated social isolation appear to have brought lifestyle factors, health behaviour and the wider determinants into sharp relief. Themes 1 and 2 highlight the impact of this for students: time spent in this period of social isolation appears to have facilitated opportunities for students to reflect on their own health behaviour; theme 3 might suggest this was catalysed by the knowledge of how LM and wider determinants influences health status.

Other published work has explored the impact of the Covid-19 pandemic on health behaviours and the perception of its importance [15]. A prospective cohort study in the UK found a reduction in fruit and vegetable consumption, reduced physical activity and increased alcohol consumption during the Spring 2020 lockdown [16]. However, these findings were tempered by a slight increase in strength training. Sleep-behaviours have been affected in different ways during lockdown. In three European countries sleep time has been shown to increase, probably due to changes in social demands and expectations that often result in sleep being sacrificed. Despite a reported increase in sleep time subjective sleep quality was shown to reduce [17]. Similar results were found in a sample of US University students [18]. Data on hand hygiene demonstrates the ability of the pandemic to stimulate behaviour change [19, 20], although this was not sustained as the pandemic progressed. This ability to catalyse behaviour change is also reflected by historical experiences with other infectious disease, such as changes to traditional burial practices and health seeking behaviour which occurred during the Ebola outbreak in 2013–2016 [21]; and compliance with Public Health measures such as hand washing, mask wearing and social distancing during the 1918–1920 influenza pandemic [22]. These are important reflections within the context of this work. Considering the sub-themes identified in theme 2, some responses indicate an intention to change, rather than changing behaviours and others demonstrate an acknowledgment of the difficulty in changing behaviour.

Living through a Public Health emergency may provide a new lens through which broader issues of health inequality are more salient and applicable, as is underscored in themes 3 and 4. The importance of translating abstract concepts into more concrete insights is reflected by the increasing importance of social accountability for medical schools [23]. Social accountability embeds institutions in the communities they serve and brings students closer to local public-health challenges, thereby allowing them to contextualise their learning.

More broadly, a shift towards experiential learning [9] is illustrated by medical schools’ moves to incorporate clinical experience into the medical degree programmes at an earlier stage [24], replacing the traditional divide between ‘pre-clinical’ and ‘clinical’ sections of the medical degree. This trend towards experiential learning is also evidence in Public Health curricula [25, 26]. Efforts to incorporate experiential learning are supported by pedagogical theory which describes it as ‘constructing knowledge and meaning from real-life experience’ [27]. For this process to occur most effectively, the learner should have some prior knowledge within which to contextualise their experience and to provide a framework for critical analysis. Thus, in our example the students’ prior knowledge came from the material taught during LMAP and reflecting on and analysing their experience of lockdown in the context of this knowledge allowed them to synthesise the insights we present in the results section. Previously published work has suggested that experiential teaching is a promising method to facilitate learning about health inequalities [28]. Health inequalities are starker now than ever, having been exacerbated by COVID-19 [29]. It is imperative that medical schools redouble their efforts to not only teach the theory underpinning health inequalities, but also practical approaches to addressing them. Our results support the idea that experiential learning, whether via simulation or real-life experience, might be a promising approach to deliver this.

Our results also suggest that the experiential learning gained through lived experience of the pandemic may provide a basis for heightened empathy. There has been previous work on the role of simulation teaching to develop empathy [30], and a review of empathy education in nursing concluded that experiential learning was the most promising form of teaching for developing this trait [31]. Moreover, in a study exploring medical students’ understanding of empathy, the students expressed a preference for experiential learning as a method of developing this trait [32]. Experiential learning may therefore be a win–win, as both an effective method of developing empathy and one which is popular with students.

One of the key aims of LM in the Imperial curriculum is to improve the health behaviours of students. It is clear in the data that students changed their behaviour in response to lockdowns, and in ways that particularly focussed on mental health. Long term health outcomes may be difficult to make real to young healthy students for whom these outcomes are remote. Living through lockdowns may bring some of these outcomes closer to home. Understanding motivation is beyond the scope of this data, but reflecting on what drives positive behaviour change in this context may provide useful insight into how these motivations could become internalised to promote longer term positive change, in line self-determination theories of learning [33].

In terms of strengths of this study it is the first study of which the authors are aware that examines at-scale the relationship between LM teaching and the COVID-19 pandemic. Among its other strengths, the response rate of 71% suggests a broad spectrum of perspectives were represented, although the generalisability of such findings to other medical schools and other pandemics is potentially limited. A further limitation is that the data were collected as part of the module evaluation, and so students may have been more likely to give positively biased answers than if they were responding to independent researchers. The data provide a snapshot of student attitudes, taken at a time when health anxiety may have been unusually high during the first UK lockdown. Importantly, the data provide no insight into how these reflections and attempts at behaviour change during lockdown impact long-term health behaviours or ongoing attitudes to LM among medical students. These are avenues for further exploration.

## Conclusion

Our results suggest that the students’ experience of the COVID-19 pandemic facilitated reflection on their own health, their empathy, and their awareness of health inequalities. We also found that lived experience might be a valuable tool for teaching LM. This can offer a double dividend since experiential learning, a form of lived experience, is also popular with students [34]. Current innovations in medical curricula offer abundant opportunity to include experiential elements, and we would advocate for this. For example, simulation teaching has been used to teach health promotion strategies for behaviour change during pandemics, with positive feedback [35]. Medical electives are another valuable opportunity for lived experience, with evidence that they contribute to reflection and the formation of professional identities [36]. Whilst the pandemic has been a terrible experience for many, it highlights the usefulness of lived experience in teaching medical students the value of concepts they traditionally do not hold in high regard, particularly in early years teaching.

## Data Availability

The datasets used and/or analysed during the current study are available from the corresponding author on reasonable request. The current data set is part of a larger, ongoing, project and so cannot be made freely available at this stage.

## Abbreviations

LM: LM
LMAP: LM and Prevention
ICSM: Imperial College School of Medicine

## References

[1] Yeh B-I, Kong ID (2013). The advent of LM. J Lifestyle Med.

[2] Mechanick JI, Kushner RF (2016). Why LM?.

[3] Nunan D, Blane DN, McCartney M (2021). Exemplary medical care or Trojan horse? An analysis of the ‘LM’movement. Br J Gen Pract.

[4] Moffat M, Sinclair HK, Cleland JA, Smith WCS, Taylor RJ (2004). Epidemiology teaching: student and tutor perceptions. Med Teach.

[5] Lyon AK, Hothersall EJ, Gillam S (2016). Teaching public health in UK medical schools: ‘things have improved: teaching no longer feels like an expensive hobby’. J Public Health.

[6] Lee JS, Xierali IM, Jaini PA, Jetpuri Z, Papa F. Medical student perception of LM and willingness to engage in lifestyle counseling: a pilot study of allopathic and osteopathic medical students. Am J of Lifestyle Med. 2021;15598276211004449:1–10. 10.1177/15598276211004449 PMC9989491 36896036

[7] Malatskey L, Essa-Hadad J, Willis TA, Rudolf MC (2019). Leading healthy lives: LM for medical students. American journal of LM.

[8] Narayan R, Rodriguez C, Araujo J, Shaqlaih A, Moss G (2013). Constructivism—constructivist learning theory.

[9] Dennick R (2016). Constructivism: reflections on twenty five years teaching the constructivist approach in medical education. Int J Med Educ.

[10] Kolb D (1984). Experiential learning: experience as the source of learning and development.

[11] Hew KF, Lo CK (2018). Flipped classroom improves student learning in health professions education: a meta-analysis. BMC Med Educ.

[12] Sandars J (2009). The use of reflection in medical education: AMEE Guide No. 44. Med Teach.

[13] Prats-Uribe A, Paredes R, Prieto-Alhambra D. Ethnicity, comorbidity, socioeconomic status, and their associations with COVID-19 infection in England: a cohort analysis of UK Biobank data. MedRxiv. 2020. 10.1101/2020.05.06.20092676.

[14] Braun V, Clarke V (2006). Using thematic analysis in psychology. Qual Res Psychol.

[15] Arora T, Grey I (2020). Health behaviour changes during COVID-19 and the potential consequences: A mini-review. J Health Psychol.

[16] Naughton F, Ward E, Khondoker M, Belderson P, Marie Minihane A, Dainty J (2021). Health behaviour change during the UK COVID-19 lockdown: findings from the first wave of the C-19 health behaviour and well-being daily tracker study. Br J Health Psychol.

[17] Blume C, Schmidt MH, Cajochen C (2020). Effects of the COVID-19 lockdown on human sleep and rest-activity rhythms. Curr Biol.

[18] Wright KP, Linton SK, Withrow D, Casiraghi L, Lanza SM, Iglesia H (2020). Sleep in university students prior to and during COVID-19 Stay-at-Home orders. Curr Biol.

[19] Moore LD, Robbins G, Quinn J, Arbogast JW (2021). The impact of COVID-19 pandemic on hand hygiene performance in hospitals. Am J Infect Control.

[20] Makhni S, Umscheid CA, Soo J, Chu V, Bartlett A, Landon E (2021). Hand hygiene compliance rate during the COVID-19 pandemic. JAMA Intern Med.

[21] Nielsen CF, Kidd S, Sillah AR, Davis E, Mermin J, Kilmarx PH (2015). Improving burial practices and cemetery management during an Ebola virus disease epidemic—Sierra Leone, 2014. MMWR Morb Mortal Wkly Rep.

[22] Balinska M, Rizzo C (2009). Behavioural responses to influenza pandemics: what do we know?. PLoS Curr.

[23] Rourke J (2018). Social accountability: a framework for medical schools to improve the health of the populations they serve. Acad Med.

[24] Başak O, Yaphe J, Spiegel W, Wilm S, Carelli F, Metsemakers JF (2009). Early clinical exposure in medical curricula across Europe: an overview. Eur J Gen Pract.

[25] Chorazy ML, Klinedinst KS (2019). Learn by doing: a model for incorporating high-impact experiential learning into an undergraduate public health curriculum. Front Public Health.

[26] Hu CXJ, Abraham A, Mitra AK, Griffiths SM (2016). The benefits of experiential learning in global Public Health. Public Health.

[27] Yardley S, Teunissen PW, Dornan T (2012). Experiential learning: transforming theory into practice. Med Teach.

[28] Billon G, Attoe C, Marshall-Tate K, Riches S, Wheildon J, Cross S (2016). Simulation training to support healthcare professionals to meet the health needs of people with intellectual disabilities. Adv Ment Health Intellect Disabil.

[29] Rimmer A (2020). Covid-19: tackling health inequalities is more urgent than ever, says new alliance. BMJ.

[30] Ter Beest H, van Bemmel M, Adriaansen M (2018). Nursing student as patient: experiential learning in a hospital simulation to improve empathy of nursing students. Scand J Caring Sci.

[31] Brunero S, Lamont S, Coates M (2010). A review of empathy education in nursing. Nurs Inq.

[32] Tavakol S, Dennick R, Tavakol M (2012). Medical students’ understanding of empathy: a phenomenological study. Med Educ.

[33] Ten Cate OTJ, Kusurkar RA, Williams GC (2011). How self-determination theory can assist our understanding of the teaching and learning processes in medical education. AMEE guide No. 59. Med Teach.

[34] Hanandeh A (2016). Can experiential learning help students' learning and improve course satisfaction?.

[35] Elder KA. Pandemic pedagogy: experiential learning illustrating risk and health communication principles. Commun Teach. 2021;36 (1):1–7.

[36] Hayashi M, Son D, Nanishi K, Eto M (2020). Long-term contribution of international electives for medical students to professional identity formation: a qualitative study. BMJ Open.

